# Dopamine D2 Receptors in Dopaminergic Neurons Modulate Performance in a Reversal Learning Task in Mice

**DOI:** 10.1523/ENEURO.0229-17.2018

**Published:** 2018-03-08

**Authors:** Jérôme Linden, Alexander S. James, Colin McDaniel, J. David Jentsch

**Affiliations:** 1 State University of New York at Binghamton, Binghamton, NY 13902; 2 University of California Los Angeles, Los Angeles, CA 90095

**Keywords:** behavioral flexibility, D2, dopamine, impulsivity, learning

## Abstract

Neuroimaging studies in animal models and human subjects have each revealed that relatively low striatal dopamine D2-like receptor binding potential is associated with poor impulse control and with vulnerability for addiction-related behaviors. These studies cannot, however, disambiguate the roles for various pools of D2 receptors found in the striatum (e.g., those expressed on medium spiny striato-pallidal neurons vs on dopamine-releasing nerve terminals) in these behavioral outcomes. To clarify the role of the latter pool, namely, D2 autoreceptors, we studied mice carrying a conditional DRD2 gene, with or without Cre-recombinase expressed under the transcriptional control of the dopamine transporter gene locus (autoDrd2-KO, *n* = 19 and controls, *n* = 21). These mice were tested for locomotor response to cocaine, and spatial reversal learning was assessed in operant conditioning chambers. As predicted, compared to control mice, autoDrd2-KO animals demonstrated heightened sensitivity to the locomotor stimulating effect of cocaine (10 mg/kg, i.p.), confirming previous research using a similar genetic model. In the spatial reversal learning task, autoDrd2-KO mice were slower to reach a learning criterion and had difficulty sustaining a prolonged nose poke response, measurements conceptually related to impaired response inhibition. Rate of learning of the initial discrimination and latencies to collect rewards, to initiate trials and to produce a response were unaffected by genetic deletion of D2 autoreceptors, discarding possible motor and motivational factors. Together, these findings confirm the role of D2 autoreceptors in reversal learning and suggest a broader involvement in behavioral inhibition mechanisms.

## Significance Statement

Because impulsivity and substance use disorder often co-occur, they may share common molecular and cellular determinants. Studies in humans and animal models alike have helped identify the bioavailability dopamine receptor D2 as a correlate of both impulsivity and predisposition for addictive behaviors. Nevertheless, these studies were not able to differentiate between the respective roles of D2 auto- and heteroreceptors. The present work provides evidence that D2 autoreceptors alone influence the performance in a task measuring impulsivity in mice, suggesting that impulsivity is not only resulting from dysfunctional striato-pallidal neurons but also might also be modulated by upstream, presynaptic events.

## Introduction

Substance use disorders are behavioral phenotypes that result from a confluence of both inherited liability factors and environmental influences that include, but are not limited to, the pharmacological effects of the drugs consumed. Indeed, experience with drugs or alcohol are not sufficient to produce a use disorder in humans, an outcome that appears to additionally depend on interindividual variability in vulnerability (Egervari et al., 2018). Identification of the bio-behavioral markers of this susceptibility could enable its measurement and aid in the design and empirical evaluation of targeted prevention strategies (Uhl, 2006).

In the search for molecular or behavioral indicators of risk for substance use disorders, dopamine D2/D3 receptors and impulsivity, as well as their mechanistic relationship, have received considerable attention. In laboratory rodents and nonhuman primates, as well as in human subjects, interindividual variation in impulsivity phenotypes have been positively correlated with alacrity to initiate drug or alcohol use, or with clinically-impairing addictions (Belin et al., 2008; Quinn et al., 2011; Jentsch et al., 2014). Moreover, dopamine D2/D3 receptors within the striatum are negatively correlated with both outcomes (Dalley et al., 2007; Lee et al., 2009; Groman et al., 2012). In other words, inherited and environmental factors that lead to relatively low forebrain D2/D3 receptors are associated with greater behavioral impulsivity (poorer impulse control) and elevated susceptibility for drug and alcohol use/use disorder.

No studies associating dopamine D2/D3 receptors to impulsivity and/or addiction liability have been able to dissect the specific subpopulation of receptors implicated. For those studies reporting a relationship between *in vivo* or *ex vivo* estimates of D2/D3 receptor binding potential or density in the striatum and impulsivity, it has been impossible to dissect the functional roles of presynaptic receptors expressed on the axon terminals of dopamine neurons from postsynaptic receptors expressed on striato-pallidal medium spiny neurons, the terminals of cortico-striatal glutamatergic axons or other neuronal populations, including striatal interneurons (Le Foll et al., 2009), although these distinct cellular subcompartments of D2/D3 receptors, in addition to having distinct functional effects on cellular physiology in the brain, may well contribute differently to impulse control phenotypes.

In animal research, the population of D2 receptors confined to dopamine neurons have themselves been linked with drug self-administration behaviors. Selective genetic depletion of D2 receptors in dopaminergic neurons augments the acquisition of cocaine self-administration behavior (de Jong et al., 2015). Moreover, higher firing activity of dopaminergic neurons, an effect that could result from D2 autoreceptor subsensitivity, is associated with heightened self-administration behavior (Marinelli et al., 2003). Based on this evidence, and the aforementioned association between addiction phenotypes and behavioral impulsivity, we hypothesized that selective reductions in D2 autoreceptors would mechanistically alter the patterns of behavioral responding in tests thought to measure aspects of the inhibitory control over impulsive and/or compulsive behaviors. Specifically, we hypothesize that deletion of the Drd2 gene will cause mice to require a greater number of trials before reaching criterion in the reversal condition. We tested this hypothesis using mice carrying *Cre-recombinase*-dependent null alleles of the Drd2 gene (which encodes the dopamine D2 receptor protein), with and without *Cre* expression driven from the *Slc6a3* (dopamine transporter encoding) locus, allowing for dopamine neuron-specific genetic deletion of D2 receptors.

## Materials and Methods

### Animals

All experimental procedures were following the National Institutes of Health Guide for Care and Use of Laboratory Animals (National Research Council, 2011). All animal procedures were performed in accordance with the State University of New York at Binghamton and University of California Los Angeles animal care committee’s policies and were approved by their respective institutional animal care and use committees.

A total of 40 male mice, aged four to six months at the start of testing, were group-housed in polycarbonate tubs with wood chip bedding; they were maintained in a humidity- and temperature-controlled vivarium (20–22°C) on a 12/12 h light/dark schedule. Animals had access to *ad libitum* food and water, except over the duration of operant testing, during when they were food-restricted to maintain them around 85% of their initial (prerestriction) body weight. No statistical methods were used to estimate the ideal sample sizes, but the numbers of animals used in the study were comparable to those reported in previous publications using similar methods. Power calculations were performed retrospectively to insure the sample size was large enough in respect of the effect size found in the statistical analyses.

B6.129S4(FVB)-*Drd2^tm1.1Mrub^*/J mice (https://www.jax.org/strain/020631) homozygous for a *Cre*-dependent, conditional allele of the dopamine D2 receptor (*Drd2*) gene in which two LoxP sites flank exon 2 were initially crossed with B6.SJL-*Slc6a3^tm1.1(cre)Bkmn^*/J mice (https://www.jax.org/strain/006660) that were hemizygous for a variant of the dopamine transporter *Slc6a3* gene directing the expression of *Cre*-recombinase (DATCre). A subset of the offspring from this cross were DATCre^+^ and carried one conditional *Drd2* allele; this progeny was mated to a DATCre^-^ mouse carrying two conditional *Drd2* alleles to produce a generation of mice bearing either the one or two conditional *Drd2* alleles and/or the DATCre allele. Mice homozygous for the conditional *Drd2* gene and hemizygous for the DATCre allele thus presented a conditional deletion of presynaptic D2 autoreceptors (AutoDrd2-KO, *n* = 19). This breeding scheme has been used in the past to produce animals with a confirmed lack of D2 autoreceptors in midbrain dopamine neurons, in the absence of broadly abnormal neurobehavioral phenotype (Bello et al., 2011). DATCre^-^ littermates, also homozygous for the conditional *Drd2* allele, were used as controls (control, *n* = 21). All animals (*N* = 40) underwent the operant reversal learning procedure, but only a subset (*N* = 28) was used in the locomotor assessment. Both experiments were conducted on male animals. Genotypes were confirmed by real-time polymerase chain reaction, using a commercial vendor (Transnetyx).

### Psychomotor response to cocaine

Locomotor response to acute cocaine exposure was tested in a subset of autoDrd2-KO (*n* = 12) and control (*n* = 16) mice after completion of the operant procedure. Locomotion was assessed in clean rat home cages (20 × 40 cm) placed into a photocell apparatus equipped with 8 infrared detectors (Omnitech Electronics, Inc.). Sessions lasted for 75 min, divided into 15 5-min time bins. Mice were first accustomed to the chamber for 30 min, during which their baseline locomotion was evaluated. At 30 min, mice were injected a single dose of cocaine (10 mg/kg, i.p.; dosages calculated as the free base weight; drug was provided by the National Institute on Drug Abuse). Cocaine-induced hyperlocomotion was then recorded for an additional 45 min.

### Reversal learning

Operant conditioning took place in a set of 8 modular mouse operant conditioning chambers (model MED-NP5M-D1; Med Associates), each equipped with an aluminum wall fitted with a food-tray, a pellet dispenser, a house light; they also contained a horizontal array of 5 nose-poke apertures on a wall on the opposite side of the box. All apertures and the food-tray were fitted with infrared beam sensors and internal lights. All chambers were enclosed in a dimly lit sound-attenuating cubicle, with white noise broadcast in the background.

#### Shaping of the operant response

On the first day of operant conditioning, animals were habituated to the operant chamber for 1 h. On the second day, all mice underwent magazine training. Pellets (14 mg Dustless Precision Pellets; Bio-Serv) were delivered after a 20-s intertrial interval (ITI); retrieval of the pellet was required before the next interval commenced. Magazine training ended after 45 min elapsed or when 50 pellets had been delivered, whichever came first. In the next phase (shaping 1), animals were trained to poke into the central aperture (hole 3 of five), on the chamber wall opposite the food tray. Trials started with illumination of this central aperture. After the expected response, the food-magazine lit up and a pellet was delivered. During the first trial, a nose poke into the central aperture led to immediate pellet delivery, while a requirement for a sustained nose poke of variable duration (0–400 ms) was imposed on subsequent trials. If an animal withdrew from the aperture before reaching the holding requirement, failure was indicated by 1-s time-out period indicated by offset of the house-light; if the mouse managed to stay in the aperture for the required period, a food pellet was delivered into the illuminated food tray. Each trial was followed by a 20-s ITI. Sessions ended after 1 h or after 100 pellets were earned, whichever came first. When a mouse managed to obtain 40 pellets in a single session, it was moved to a similar procedure that differed only in that the nose poke duration requirements were extended to 600 ms (shaping 2). After reaching the 40 pellets criterion again, the response hold requirements were set to vary between 400 and 600 ms (shaping 3).

#### Acquisition

After initial training of the variable duration nose poke response to the central aperture, the acquisition of a simple spatial discrimination commenced. In these sessions, each trial was again initiated by a sustained, variable duration nose poke in aperture 3 (200–600 ms). Once this observing response was completed, the flanking apertures (holes 2 and 4) were illuminated for up to 30 s. Selected in a pseudorandom fashion for each mouse, subsequent responses (this time a simple, not-sustained nose poke) into the right or left hole (counterbalanced across genotypes) were rewarded with a food pellet, while responses in the other aperture led to a 3-s time-out period (signaled with darkness). A failure to respond within the 30-s period of illumination was counted as an omission. Each trial was followed by a 3-s ITI. Daily sessions lasted for 60 min or until the acquisition criterion was met. This acquisition criterion consisted of performing 16 correct responses in a moving window of 20 trials.

#### Reversal

Reversal learning was similar to the acquisition phase, except mice had to respond in the opposite aperture to the one previously reinforced. Reversal was achieved once an animal performed 16 correct responses out of 20 trials. Sessions lasted 60 min, or until the criterion was met.

### Statistics

The numbers of trials to reach learning criterion were compared using a one-tail Student’s *t* test. All other measures were compared using mixed-model ANOVAs (the Greenhouse–Geisser correction was used when data did not meet the sphericity criterion). All statistical analyses were performed with Statistica 13.0 or SPSS 23.0. Results were considered significant at *p* < 0.05. A summary of all the statistical analyses can be found in Table 1.

**Table 1.** Summary of statistical analyses

	Measure	Factor(s)	Data structure	Type of test	Statistical value	*p* value
a	Cocaine locomotion (time bins)	Time	Normal	ANOVA	*F* = 30.891	<0.001
		Time × genotype	Normal	ANOVA	*F* = 5.584	<0.001
		Genotype	Normal	ANOVA	*F* = 12.049	0.002
b	Cocaine locomotion (pre- vs postexposure)	Cocaine	Normal	ANOVA	*F* = 40.545	<0.001
		Cocaine × genotype	Normal	ANOVA	*F* = 9.936	0.004
		Genotype	Normal	ANOVA	*F* = 11.693	0.002
c	Shaping of the operant response	Stage	Non-normal	ANOVA	*F* = 18.680	<0.001
		Stage × genotype	Non-normal	ANOVA	*F* = 0.181	0.812
		Genotype	Non-normal	ANOVA	*F* = 1.308	0.265
d	Trials to criterion	Reversal	Non-normal	One-tailed *t* test	*t* = 1.695	0.051
		Phase	Non-normal	ANOVA	*F* = 31.848	<0.001
		Phase × genotype	Non-normal	ANOVA	*F* = 1.264	0.268
		Genotype	Non-normal	ANOVA	*F* = 3.552	0.067
e	Errors to criterion	Phase	Non-normal	ANOVA	*F* = 29.65	<0.001
		Phase × genotype	Non-normal	ANOVA	*F* = 0.678	0.416
		Genotype	Non-normal	ANOVA	*F* = 2.636	0.113
f	Omissions per trial	Phase	Non-normal	ANOVA	*F* = 3.370	0.074
		Phase × genotype	Non-normal	ANOVA	*F* = 0.143	0.143
		Genotype	Non-normal	ANOVA	*F* = 1.640	0.208
g	Trial initiation latency	Phase	Non-normal	ANOVA	*F* = 9.548	0.003
		Phase × genotype	Non-normal	ANOVA	*F* = 0.239	0.63
		Genotype	Non-normal	ANOVA	*F* = 0.0485	0.49
h	Pellet retrieval latency	Phase	Non-normal	ANOVA	*F* = 5.385	0.026
		Phase × genotype	Non-normal	ANOVA	*F* = 1.937	0.172
		Genotype	Non-normal	ANOVA	*F* = 0.441	0.51
i	Failed/successful initiation attempts	Hold requirement	Non-normal	ANOVA	*F* = 77.362	<0.001
		Hold requirement × genotype	Non-normal	ANOVA	*F* = 7.343	0.008
		Genotype	Non-normal	ANOVA	*F* = 8.578	0.006

Data distribution, test employed, statistic, and *p* value are detailed for each behavioral index and each factor.

To demonstrate that nonsignificant effects could not simply result from underpowered analyses and to substantiate the reality of significant effects, we performed additional analyses, calculating effect size, *post hoc* power (or observed power) and ideal sample size for each behavioral index and each tested factor. We also provided an interpretation of effect sizes (Cohen, 1988). These analyses were performed with G*Power 3 (Faul et al., 2007) and are summarized in Table 2.

**Table 2.** Power and sample size analyses

	Measure	Factor(s)	*p* value	Effect size			*Post hoc*power	Sample size	
				Partial *η^2^*	Cohen’s *f*	Interpretation		Ideal	Actual
a	Cocaine locomotion (time bins)	Time	<0.001	0.543	1.09	Large	1	4	28
		Genotype	<0.001	0.317	0.681	Large	0.964	15	28
		Time × genotype	0.002	0.226	0.54	Medium	0.916	10	28
b	Cocaine locomotion (pre- vs postexposure)	Time	<0.001	0.609	1.248	Large	1	6	28
		Genotype	0.004	0.31	0.67	Large	0.855	16	28
		Time × genotype	0.002	0.274	0.614	Large	0.908	4	28
c	Shaping of the operant response	Stage	<0.001	0.679	0.901	Large	1	4	27
		Genotype	0.812	0.054	0.239	Small	N/A	114	27
		Stage × genotype	0.265	0.008	0.09	Null	N/A	124	27
d	Trials to criterion	Phase	<0.001	0.456	0.915	Large	N/A	6	40
		Genotype	0.268	0.085	0.305	Small	1	54	40
		Phase × genotype	0.067	0.032	0.182	Small	N/A	66	40
e	Errors to criterion	Phase	<0.001	0.438	0.883	Large	1	8	40
		Genotype	0.416	0.065	0.264	Small	N/A	64	40
		Phase × genotype	0.113	0.018	0.135	Null	N/A	202	40
f	Omissions per trial	Phase	0.074	0.081	0.297	Small	N/A	12	40
		Genotype	0.143	0.041	0.207	Small	N/A	166	40
		Phase × genotype	0.208	0.056	0.244	Small	N/A	18	40
g	Trial initiation latency	Phase	0.003	0.205	0.508	Medium	0.853	22	40
		Genotype	0.63	0.013	0.115	Null	N/A	236	40
		Phase × genotype	0.49	0.06	0.253	Small	N/A	78	40
h	Pellet retrieval latency	Phase	0.026	0.015	0.124	Null	0.619	192	40
		Genotype	0.172	0.011	0.105	Null	N/A	448	40
		Phase × genotype	0.51	0.048	0.225	Small	N/A	60	40
i	Failed/successful initiation attempts	Hold requirement	<0.001	0.671	1.428	Large	1	4	40
		Genotype	0.008	0.184	0.475	Medium	0.784	30	40
		Hold requirement × genotype	0.006	0.162	0.44	Medium	0.814	12	40

*Post hoc* power and effect size are reported for each behavioral measure, as well as a comparison between ideal and actual sample sizes.

## Results

### Psychomotor response to cocaine

**Figure 1.**
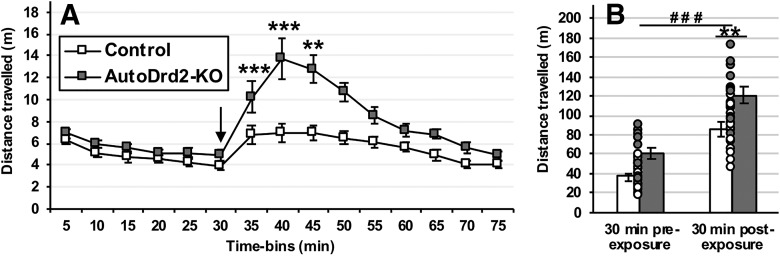
Locomotor response to cocaine. While both groups expressed cocaine-increased locomotion after cocaine injection (arrow), autoDrd2-KO mice showed an exaggerated response compared to littermate controls, as shown by significant differences between genotypes appearing during the 35-, 40-, and 45-min time bins (**p = 0.01, ***p < 0.001; ***A***). When comparing locomotion during the 30-min pre- vs postexposure to cocaine, there was an overall significant effect of the drug (###*p* < 0.001), as well as a significant difference between genotypes during the postexposure period (**p < 0.01; ***B***). Each dot represents a single data point. Bars show group means ± SEM.

The 75-minute locomotor activity session was divided in 15 time bins, and the average distance traveled was analyzed using a repeated measures ANOVA, with time as the within-subject factor and genotype as the between-subjects factor. The ANOVA yielded a significant main effect of genotype (*F*_(1,26)_ = 12.049; *p* = 0.002^a^), a significant main effect of time (*F*_(14,364)_ = 30.891; *p* < 0.001^a^) and a significant genotype × time interaction (*F*_(14,364)_ = 7.584; *p* < 0.001)^a^. Using Bonferroni-corrected *post hoc* tests, we first sought to characterize the locomotion-stimulating effect of cocaine in both control and autoDrd2-KO animals separately. Both groups appeared to experience the stimulating effect of cocaine: in the control group, the traveled distance increased significantly between the 30-, 40-, and 45-min time bins (all *p* < 0.005), while in the autoDrd2-KO group the increase extended through the 50- and 55-min time bins (all *p* < 0.001). We then compared experimental and control groups independently for each time bin. The *post hoc* tests revealed a significant difference between genotypes at 40 (*p* < 0.001), 45 (*p* < 0.001), and 50 min (*p* = 0.01; 10, 15, and 20 min after cocaine injection, respectively; Fig. 1*A*). To provide a more synthetic approach and highlight the difference in cocaine activation across genotypes, we also compared the total traveled distance during the first 30 min before cocaine injection (pre-exposure) to the 30-min following cocaine injection (postexposure) using a mixed-model ANOVA with the cocaine (pre- vs postexposure) as the within-subject factor and genotype as between-subjects factor. The analysis revealed a significant main effect of genotype (*F*_(1,26)_ = 11.693; *p* = 0.002^b^) and of cocaine (*F*_(1,26)_ = 40.545; *p* < 0.001^b^), as well as a significant cocaine × genotype interaction (*F*_(1,26)_ = 9.836; *p* = 0.004^b^). Furthermore, a Bonferroni-corrected *post hoc* comparison showed that: (1) baseline pre-exposure locomotion was comparable between genotypes (*p* = 0.144), (2) both autoDrd2-KO and control mice saw a significant increase in locomotor activity after cocaine injection (*p* < 0.001 and *p* = 0.02, respectively), and (3) autoDrd2-KO displayed a significantly exacerbated locomotor response to cocaine (*p* = 0.001; Fig. 1*B*).

In each case, autoDrd2-KO mice exhibiting a significantly greater locomotor response to cocaine than littermate controls, as expected (Bello et al., 2011; Koulchitsky et al., 2016).

### Shaping of the operant response

**Figure 2.**
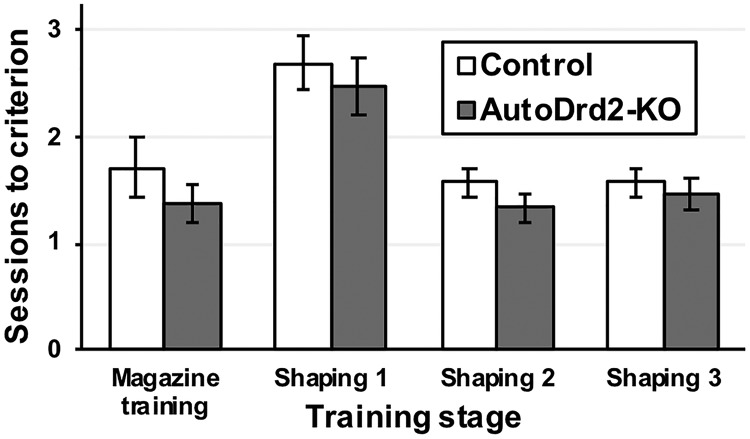
Shaping of the operant response. Genotype had no effect on the total number of sessions needed to learn the operant response (*p* < 0.265). However, when both groups were considered together, the first stage of shaping (shaping 1) took significantly longer than any other stage (all *p* < 0.01). Bars show group means ± SEM.

Because dopamine neurons are a key component in reward-based learning, there is a possibility that manipulations altering dopaminergic transmission also interfered with reward-based learning abilities. To explore this possibility, we compared the number of sessions to reach learning criterion in each training stage (magazine training; shaping 1, 2, and 3) with a mixed-model ANOVA, with the stage as within-subject factor and genotype as between subject factor in a subset of autoDrd2-KO (*n* = 13) and control mice (*n* = 14). The ANOVA showed that there was a significant training stage main effect (*F*_(3,69)_ = 18.680; *p* < 0.001^c^), but there was no main effect of genotype (*F*_(1,23)_ = 1.308; *p* = 0.265^c^) nor any genotype × stage interaction (*F*_(3,69)_ = 0.181; *p* = 0.812^c^) regarding the number of sessions needed to learn the response. A Bonferroni-corrected *post hoc* test indicated that animals took longer in the initial stage, during which they had to first learn the operant response (shaping 1), than in either subsequent stage (all *p* < 0.01; Fig. 2).

### Reversal learning

**Figure 3.**
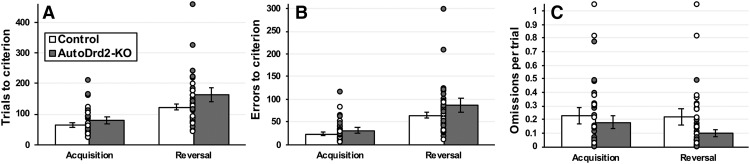
Trials to criterion, errors to criterion and omission rates. Although there was no effect of genotype on the number of trials needed to reach criterion, testing the specific hypothesis of a slower performance in autoDrd2-KO during the reversal phase with a *t* test returned a probability closely approaching significance level (*p* = 0.051; ***A***). There was no statistically significant difference between the groups in the total numbers of errors committed (***B***) or in the number of omissions per trial (***C***). Each dot represents a single data point. Bars show group means ± SEM.

The reversal learning test produces a large set of behavioral measures, each collected at two training stages: acquisition and reversal. However, the a priori hypothesis tested in these studies was that reversal learning would be impaired in autoDrd2-KO (*n* = 19), compared with wild-type (*n* = 21), mice. This hypothesis was evaluated using a one-tailed *t* test, revealing a statistical trend for autoDrd2-KO mice to require more trials than wild-type controls to reach criterion performance during reversal learning (*t*_(39)_ = 1.696, *p* = 0.051; Fig. 3*A*).

The larger dataset was next evaluated using repeated measures ANOVAs, with the genotype as between-subject factor and testing phase as the within-subject factor. Fig. 3*A* exhibits the pattern of effects when trials to reach criterion in the initial acquisition and subsequent reversal phase were evaluated in both genotype groups. These analyses revealed a main effect o*f* testing phase, with animals requiring more trials, on average, to reach criterion during reversal learning, compared to initial acquisition (*F*_(1,38)_ = 31.8; *p* < 0.001^d^). ANOVA also revealed a trend for a main effect of genotype (*F*_(1,38)_ = 3.6; *p* = 0.067^d^), but the genotype × testing phase interaction was not significant (*F*_(1,38)_ = 1.3; *p* = 0.268^d^). In the analyses of the total number of erroneous responses (nose pokes in the nonreinforced aperture), the main effect of genotype (*F*_(1,38)_ = 2.636; *p* = 0.113^e^) was not significant, but there was a significant effect o*f* testing phase (*F*_(1,38)_ = 29.95; *p* < 0.001^e^); the interaction between the two variables was not significant (*F*_(1,38)_ = 0.678; *p* = 0.416^e^; Fig. 3*B*). Finally, analyses of the average number of omissions occurring per trial did not reveal any main effect of genotype (*F*_(1,38)_ = 1.64; *p* = 0.208^f^) or any genotype × testing phase interaction (*F*_(1,38)_ = 2.238; *p* = 0.143^f^); the main effect o*f* testing phase was not significant, although there was a trend to omit more trial across testing phases (*F*_(1,38)_ = 3.37; *p* = 0.074^f^; Fig. 3*C*).

**Figure 4.**
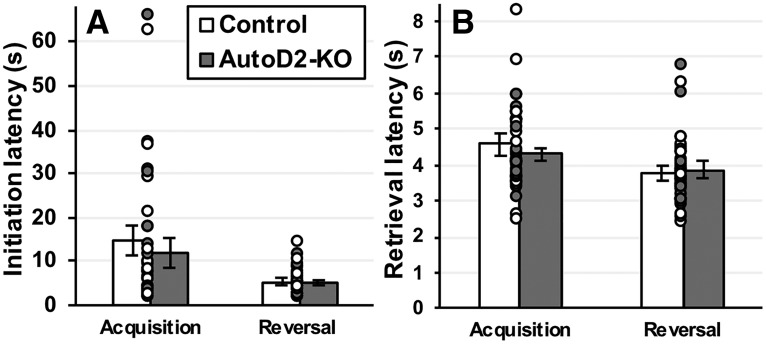
Latencies to initiate trials and collect food rewards. When compared across genotypes, both the latency to initiate trials (***A***) and the latency to retrieve food rewards (***B***) were unaffected by genotype. Each dot represents a single data point. Bars show group means ± SEM.

Analyses next focused on the average trial initiation times and pellet retrieval latencies, to evaluate possible genotype effects on motor or motivational vigor. AutoDrd2-KO did not differ from wild-type control mice on any of these measures. Trial initiation times were systematically affected by testing phase (*F*_(1,38)_ = 9.5; *p* = 0.003^g^), but not by genotype (*F*_(1,38)_ = 0.5; *p* = 0.49^g^); no genotype × testing phase interaction was detected (*F*_(1,38)_ = 0.2; *p* = 0.63^g^; Fig. 4*A*). Similarly, reward retrieval latencies were affected by testing phase (*F*_(1,38)_ = 5.4; *p* = 0.026^h^) but not genotype (*F*_(1,38)_ = 0.4; *p* = 0.510^h^), nor was there a genotype × testing phase interaction (*F*_(1,38)_ = 1.9; *p* = 0.172^h^; Fig. 4*B*).

**Figure 5.**
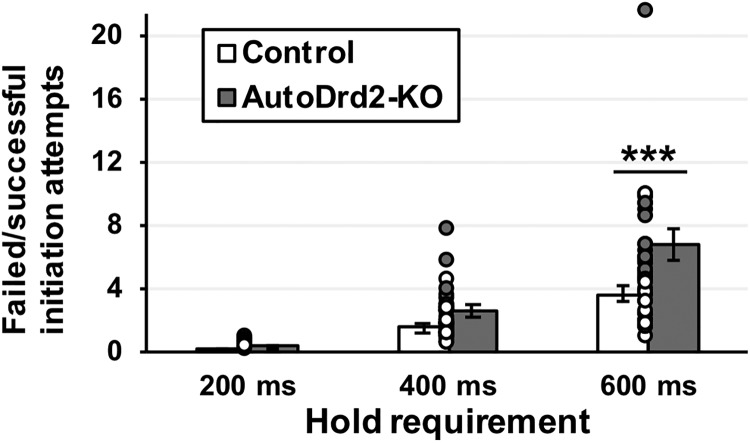
Ratio of failed to successful attempts to perform a sustain nose-poking response. Overall, autoDrd2-KO mice showed an exacerbated failure rate compared to controls (*p* = 0.013); this effect was driven mostly by the 600-ms hold requirement (***p = 0.001). Each dot represents a single data point. Bars show group means ± SEM.

Using a 5-choice serial reaction time task (5CSRTT), Dalley et al. (2007) showed that rats exhibiting elevated levels of anticipatory responding (nose-pokes made before a target stimulus was delivered) exhibit a lower availability of D2-like receptors in the ventral striatum. In the current task, mice must make a sustained response at the central hole to trigger trial onset, and difficulty with doing so may reflect a response inhibition or waiting deficit. The average number of times per trial that mice withdrew their snout early from the central hole were evaluated as a function of genotype and the programmed hold duration (which varied across trials). ANOVA revealed a main effect of genotype (*F*_(1,38)_ = 8.578; *p* = 0.006^i^), a main effect of hold requirement (*F*_(2,76)_ = 77.362; *p* < 0.001^i^) and, after applying the Greenhouse–Geisser correction, a significant interaction between genotype and hold requirement (*F*_(2,76)_ = 7.34; *p* = 0.008^i^). A Bonferroni-corrected *post hoc* test revealed a significant difference between AutoDrd2-KO and control mice in the ability to successfully wait until the hold requirement was completed when that duration was set at 600 ms (*p* = 0.001), but not when the hold duration was set at either 200 ms (*p* > 0.999) or 400 ms (*p* > 0.999; Fig. 5).

## Discussion

Dopamine D2 receptors have been implicated in the pathophysiology of various psychiatric disorders, including impulse control conditions and substance use disorders, but very few studies have examined the specific contributions of pre- vs postsynaptic D2 receptors in relevant behavioral endophenotypes. Based on the observation of a strong relationship between individual-level variation in striatal dopamine D2-like receptor complement and reversal learning (Groman et al., 2011, 2014, 2016; Izquierdo and Jentsch, 2012), we tested whether the fraction localized to presynaptic dopaminergic neuronal terminals contributed to this effect. We found that autoDrd2-KO mice lacking D2 autoreceptors tend to display relatively poorer reversal learning capacities, although this effect is made ambiguous by the unchanged number of incorrect responses. More significantly, they also exhibited difficulty with completing a sustained nose-poke response that required the ability to wait to obtain reward. Both outcomes suggest that presynaptic D2 receptors have a role in influencing mechanisms such as behavioral or response inhibition.

To compare our results with this genetic model with past research, we also evaluated the locomotor responses to an acute dose of cocaine in autoDrd2-KO and control littermate animals. AutoDrd2-KO mice showed potentiated cocaine-induced hyperlocomotion. Previous studies that used the same construct found similar results linking excess dopamine release caused by the lack of D2 autoreceptor-mediated feedback inhibition to greater locomotor responses to this stimulant drug of abuse (Bello et al., 2011; Holroyd et al., 2015).

### Relationships between dopamine and different forms of behavioral control

Reversal learning tests, including the variant used here, measure multiple psychological/behavioral processes, including (but not limited to) sensorimotor abilities, reinforcement learning, incentive motivation and inhibitory control over a pre-potent response. Given that this test has been shown to be sensitive to D2 receptor function (Groman et al., 2009; Izquierdo and Jentsch, 2012; Jentsch et al., 2014), we hypothesized that deletion of presynaptic D2 receptors would impede the ability to learn the new reinforcement contingencies when reversal occurred. We identified a pronounced trend in autoDrd2-KO animals to require more trials before reaching criterion after reversal of learning contingencies, although this effect did not reach a conventional significance level. Additional analyses of the results gathered showed genotype effects on another response inhibition-related index, namely the ability to perform sustained duration observing response to initiate trials. In the current studies, autoDrd2-KO mice displayed a clear difficulty with maintaining this response for longer, but not shorter, durations. The absence of motor hyperactivity at baseline in the locomotor test, as well as no differences in the response times associated with trial initiation or pellet retrieval suggest that the difficulty maintaining the sustained duration observing response is not simply a hyperactivity phenotype.

Whereas difficulty to learn a reversed contingency is considered as “action impulsivity,” inability to perform a sustain response is more closely linked to premature responding, which operationally reflects “waiting impulsivity” (Robinson et al., 2009; Voon et al., 2014). Although both share partially common neural circuits, evidence suggest that the D2 receptors exert have an opposite effect on action and waiting impulsivity, as the D2/D3 agonist quinpirole impairs reversal learning but reduces premature responding (Boulougouris et al., 2009; Fernando et al., 2012). The complex role of D2 receptors on impulsivity might explain why deletion of the D2 autoreceptors only moderately affected reversal learning while more severely impairing execution of long sustained responses in the present study.

Reversal learning has been repeatedly used as a measure of behavioral flexibility in rodents, monkeys and human subjects, possibly reflecting the capacity for inhibitory control. However, the results of pharmacological studies have sometimes produced mixed results regarding the relationship of this phenotype to dopaminergic systems. Manipulation of dopamine release with psychostimulant drugs – such as cocaine or *D-*amphetamine - leads to inconsistent results within the reversal learning paradigm. In rats, reversal learning is compromised by acute administration of *D-*amphetamine (Idris et al., 2005), while not being affected by methylphenidate (Seu and Jentsch, 2009; Cheng and Li, 2013). Acute administration of cocaine impairs reversal learning in monkeys (Jentsch et al., 2002). In humans, small doses of cocaine improve reversal performance (Spronk et al., 2016), while methylphenidate is reported to produce opposite effects on reversal learning depending on working memory load and trait impulsiveness (Clatworthy et al., 2009; van der Schaaf et al., 2013). The effects of drugs interacting with dopamine on behavioral control are not always consistent across studies, though incongruences might result from the nonspecific action of cocaine, amphetamine and methylphenidate, as all of them also interact with other monoaminergic neurotransmitters, mainly serotonin and norepinephrine (Segal and Kuczenski, 1997; Andrews and Lucki, 2001).

Nevertheless, inconsistencies are also found in studies of genetically-engineered mice lacking the dopamine transporter (resulting in a prolonged presence of dopamine in the synapse); this genetic model has been associated with either improvement (Milienne-Petiot et al., 2017) or impairment (Del’Guidice et al., 2014) in reversal learning, although in both cases the change was rather modest. One explanation for the ambiguous effects of dopaminergic manipulations might reside in the dual nature of reversal learning, which requires subjects to not only to inhibit a pre-potent response but also to concurrently learn a new association. It is also important to mention that although many studies have aimed to assess reversal learning, they do so using tasks that vary greatly in their procedural aspects (training regimen, schedule of reinforcement, type/number of discriminanda, etc.). This might make the comparison between this task and our own results uncertain and lead to some of the reported inconsistencies.

The role of D2 receptors in behavioral control has been better characterized by the use of specific pharmacological agents. D2/D3 agonist quinpirole impaired reversal learning in rats, but did not affect acquisition or retention of the operant response (Boulougouris et al., 2009). Similarly, microinfusions of quinpirole in the nucleus accumbens impaired. Reversal learning performance (but not set-shifting) in rats (Haluk and Floresco, 2009). Human subjects who received bromocriptine, a D2 agonist, also showed decreased performance in a probabilistic reversal learning task (Mehta et al., 2001), though the D3-preferring agonist, pramiprexole, did not alter perseverative responses in healthy volunteers in a similar test (Ersche et al., 2011). Seemingly paradoxically, blockade of D2 receptors is also associated with poor reversal learning abilities. The selective D2 antagonist eticlopride, when infused into the orbitofrontal cortex, disrupted performance of a reaction-time task after reversal of reinforcement contingencies (Calaminus and Hauber, 2008). In nonhuman primates (vervet monkeys), D2/D3 receptor antagonist raclopride decreased reversal performance, without affecting discrimination learning per se (Lee et al., 2007). Finally, D2 receptor blockade with sulpiride also hindered reversal learning in healthy humans (Janssen et al., 2015).

Qualitatively similar impairments in reversal learning associated with either activation or blockade of D2 subtype receptors might be explained by the role dopamine plays in inhibitory control, taking into account the substantial range of individual variation in the magnitude of transmission. Recently, an elegant neuroimaging study showed that subjects with higher dopamine synthesis capacity exhibited better learning in response to rewards in a deterministic reversal learning task but also exhibited impaired reversal performance in response to a D2 agonist, while subjects with lower dopamine synthesis capacity learned more from punishment and showed improved performance in response to a D2 agonist. One interpretation of these results is that there exists a curvilinear relationship between dopaminergic transmission and reversal learning (Cools et al., 2009); in such a model, the role for high-affinity D2-type receptors, located both pre- and postsynaptically, in affecting reversal learning varies in a manner influenced by subjects’ trait dopamine synthesis and release, as well as by the task/testing conditions that could independently influence dopaminergic activity.

Our task also allowed for the measurement of the ability to perform a sustained response which may, in a simpler way, reflect the capacity for response or behavioral inhibition. It is somewhat similar in principle to the ability to wait in either a differential reinforcement of low-rate responding (DRL) schedule or in the 5CSRTT. In the DRL test, animals are reinforced for producing an operant response only if it follows a period of time of preset duration during which no response was made; it is thus regarded as a measure of the ability to wait/defer a reward-eliciting response (Ferster and Skinner, 1957; Stoffel and Cunningham, 2008; Jentsch et al., 2014). Treatments with psychostimulant drugs elicit an impulsive pattern of responding in the DRL (Wenger and Wright, 1990; Lobarinas and Falk, 1999; Liao and Cheng, 2005; Cheng and Liao, 2007). Similarly, the number of responses that anticipate target delivery in the 5CSRTT is also thought to measure a similar waiting construct (Robbins, 2002; Sanchez-Roige et al., 2012). Similar to the DRL schedule, cocaine and *D-*amphetamine also increase impulsive responding in the 5CSRTT in mice (Loos et al., 2010) or rats (Grottick and Higgins, 2002; Van Gaalen et al., 2006; Pattij et al., 2007; Paterson et al., 2011; Baarendse and Vanderschuren, 2012). The ability to wait may exhibit a more consistent pattern of change (relative to reversal learning) following dopaminergic manipulations, suggesting it may have utility in mechanistic studies examining the consequences of genetic and/or pharmacological manipulations of the type used here.

### Specific roles for pre- and postsynaptic D2 receptors in behavioral control

As noted above, lower availability, levels and/or functionality of striatal dopaminergic D2 receptors have been consistently linked to impulsive behavioral phenotypes in laboratory rodents and human or nonhuman primates (Kruzich and Grandy, 2004; Kruzich et al., 2006; Dalley et al., 2007; Boulougouris et al., 2009; De Steno and Schmauss, 2009; Buckholtz et al., 2010; Groman et al., 2011; Laughlin et al., 2011; Morita et al., 2016). D2 receptors are located within multiple cellular compartments within the striatum, including postsynaptic medium spiny neurons that project to the pallidum, as well as on dopamine-releasing terminals where they regulate dopamine synthesis and release (Jentsch and Roth, 2000). Past studies linking impulsivity with D2-like receptors, measured in tissue homogenates or with positron emission tomography, were unable to differentiate these two populations because the ligands used in pharmacological studies target both pre- and postsynaptic receptors and have affinity for both the D2 and D3 receptor subtypes. Our studies suggest that the D2 subtype located on dopamine neurons contributes, at least in part, to the relationship between impulse control phenotypes and the dopamine D2-like receptor complements measured in previous studies.

In addition to the D2 family of dopamine receptors, D1 receptors have been characterized as exerting complementary role in transmitting dopaminergic neuronal signals. Inhibition of nonrelevant motor patterns relies on a fine balance between tonic and phasic dopamine release. In one theory, tonic dopamine acts on high-affinity postsynaptic D2 receptors, while phasic bursts of dopamine energize behavioral outputs through activation of lower affinity D1 receptors (Baik, 2013; Volkow and Morales, 2015; Soares-Cunha et al., 2016). Because deletion of D2 autoreceptors is predicted to disinhibit phasic dopamine release, it is likely that both postsynaptic D1 and D2 are anomalously activated in autoDrd2-KO mice, but that the resulting cellular effects produce dissociable modulation of behavioral control. Direct silencing of D1 versus D2-expressing medium spiny neurons produce different effects on reinforcement learning and inhibitory control, with only modulation of D2-expressing striato-pallidal neurons selectively impairing reversal learning (Yawata et al., 2012). These findings are consistent with the notion that dopamine D2 receptors, and their actions on indirect pathway output neurons of the striatum, may more specifically relate to the selection of a single appropriate response (and consequently, to the inhibition of others; Keeler et al., 2014).

## Conclusions

The present study extends on the substantial evidence linking impulse control phenotypes to brain dopamine D2-like receptors (Jentsch et al., 2014). These studies suggest that the relationship between low dopamine D2 receptor complement and impaired behavioral flexibility in a reversal learning task (Groman et al., 2014) is mediated, at least in part, by the fraction of D2 receptors localized to dopaminergic nerve terminals, a finding consistent with the link between low midbrain D2 receptor binding potential and impulsivity in humans (Buckholtz et al., 2010). By contrast, a recent positron emission tomography study in rats suggested that relatively higher D3-specific binding in the midbrain dopaminergic nuclei is also associated with behavioral inflexibility (Groman et al., 2016). These studies are therefore highlighting dissociable contributions of specific subtypes of dopamine D2 receptors and of the cellular compartments in which they are expressed. Only through systematic analyses of the role for D2 versus D3 receptors in pre- and postsynaptic circuits can a thorough characterization of their effects be provided. Given the importance of impulse control, and its modulation by D2 receptors, to drug addiction (Jentsch and Pennington, 2014), the mechanistic details of these modulatory effects are important to the design and implementation of rational strategies for enhancing inhibitory self-control over the impulsive (and perhaps compulsive) aspects of clinically-impairing substance use.

## Synthesis

Reviewing Editor: Christian Lüscher, University of Geneva Decisions are customarily a result of the Reviewing Editor and the peer reviewers coming together and discussing their recommendations until a consensus is reached. When revisions are invited, a fact-based synthesis statement explaining their decision and outlining what is needed to prepare a revision will be listed below. The following reviewer(s) agreed to reveal their identity: Marc Exton-McGuinness

There is one point that still needs to addressed: in the rebuttal the authors argue that running more animals in order to obtain sufficient power is p-hacking ; however, it is the opinion of the reviewer that this statement is vastly overstated.

If this is in fact the case the authors should clearly state the power calculation within the manuscript and provide information on how the exact effect size and necessary sample size was determined. Most power calculations are based on an estimated effect size from previous experiments, so it is unclear how the authors could be certain that they have sufficient statistical power in this case.

Also there are some minor editing issues. In the methods it may be worth changing ‘aperture 1,2,3’ to 'stage 1,2,3' to match the results analysis. This also prevents any confusion with 'hole 1-5' as I assume all these stages were in the same hole (hole 3).

## References

[B1] Andrews CM, Lucki I (2001) Effects of cocaine on extracellular dopamine and serotonin levels in the nucleus accumbens. Psychopharmacology (Berl) 155:221–229. 10.1007/s002130100704 11432683

[B2] Baarendse PJJ, Vanderschuren LJMJ (2012) Dissociable effects of monoamine reuptake inhibitors on distinct forms of impulsive behavior in rats. Psychopharmacology (Berl) 219:313–326. 10.1007/s00213-011-2576-x 22134476 PMC3249190

[B3] Baik JH (2013) Dopamine signaling in reward-related behaviors. Front Neural Circuits 7:152. 10.3389/fncir.2013.00152 24130517 PMC3795306

[B4] Belin D, Mar AC, Dalley JW, Robbins TW, Everitt BJ (2008) High impulsivity predicts the switch to compulsive cocaine-taking. Science 320:1352–1355. 10.1126/science.1158136 18535246 PMC2478705

[B5] Bello EP, Mateo Y, Gelman DM, Noaín D, Shin JH, Low MJ, Alvarez VA, Lovinger DM, Rubinstein M (2011) Cocaine supersensitivity and enhanced motivation for reward in mice lacking dopamine D2 autoreceptors. Nat Neurosci 14:1033–1038. 10.1038/nn.2862 21743470 PMC3175737

[B6] Boulougouris V, Castañé A, Robbins TW (2009) Dopamine D2/D3 receptor agonist quinpirole impairs spatial reversal learning in rats: investigation of D3 receptor involvement in persistent behavior. Psychopharmacology (Berl) 202:611–620. 10.1007/s00213-008-1341-2 18836703

[B7] Buckholtz JW, Treadway MT, Cowan RL, Woodward ND, Li R, Ansari MS, Baldwin RM, Schwartzman AN, Shelby ES, Smith CE, Kessler RM, Zald DH (2010) Dopaminergic network differences in human impulsivity. Science 329:532. 10.1126/science.1185778 20671181 PMC3161413

[B8] Calaminus C, Hauber W (2008) Guidance of instrumental behavior under reversal conditions requires dopamine D1 and D2 receptor activation in the orbitofrontal cortex. Neuroscience 154:1195–1204. 10.1016/j.neuroscience.2008.04.046 18538938

[B9] Cheng JT, Li JS (2013) Intra-orbitofrontal cortex injection of haloperidol removes the beneficial effect of methylphenidate on reversal learning of spontaneously hypertensive rats in an attentional set-shifting task. Behav Brain Res 239:148–154. 10.1016/j.bbr.2012.11.006 23159707

[B10] Cheng RK, Liao RM (2007) Dopamine receptor antagonists reverse amphetamine-induced behavioral alteration on a differential reinforcement for low-rate (DRL) operant task in the rat. Chin J Physiol 50:77–88. 17608145

[B11] Clatworthy PL, Lewis SJG, Brichard L, Hong YT, Izquierdo D, Clark L, Cools R, Aigbirhio FI, Baron JC, Fryer TD, Robbins TW (2009) Dopamine release in dissociable striatal subregions predicts the different effects of oral methylphenidate on reversal learning and spatial working memory. J Neurosci 29:4690–4696. 10.1523/JNEUROSCI.3266-08.2009 19369539 PMC6665353

[B12] Cohen J (1988) Statistical power analysis for the behavioral sciences. Hillsdale, NJ: Lawrence Earlbaum Associates.

[B13] Cools R, Frank MJ, Gibbs SE, Miyakawa A, Jagust W, D’Esposito M (2009) Striatal dopamine predicts outcome-specific reversal learning and its sensitivity to dopaminergic drug administration. J Neurosci 29:1538–1543. 10.1523/JNEUROSCI.4467-08.2009 19193900 PMC2940719

[B14] Dalley JW, Fryer TD, Brichard L, Robinson ESJ, Theobald DEH, Laane K, Pena Y, Murphy ER, Shah Y, Probst K, Abakumova I, Aigbirhio FI, Richards HK, Hong Y, Baron JC, Everitt BJ, Robbins TW (2007) Nucleus accumbens D2/3 receptors predict trait impulsivity and cocaine reinforcement. Science 315:1267–1270. 17332411 10.1126/science.1137073 PMC1892797

[B15] de Jong JW, Roelofs TJM, Mol FMU, Hillen AEJ, Meijboom KE, Luijendijk MCM, van der Eerden HAM, Garner KM, Vanderschuren LJMJ, Adan RAH (2015) Reducing ventral tegmental dopamine D2 receptor expression selectively boosts incentive motivation. Neuropsychopharmacology 40:2085–2095. 10.1038/npp.2015.60 25735756 PMC4613606

[B16] De Steno DA, Schmauss C (2009) A role for dopamine D2 receptors in reversal learning. Neuroscience 162:118–127. 10.1016/j.neuroscience.2009.04.052 19401217 PMC2873774

[B17] Del’Guidice T, Lemasson M, Etiévant A, Manta S, Magno LAV, Escoffier G, Roman FS, Beaulieu J-M (2014) Dissociations between cognitive and motor effects of psychostimulants and atomoxetine in hyperactive DAT-KO mice. Psychopharmacology (Berl) 231:109–122. 10.1007/s00213-013-3212-8 23912772

[B18] Egervari G, Ciccocioppo R, Jentsch JD, Hurd YL (2018) Shaping vulnerability to addiction – the contribution of behavior, neural circuits and molecular mechanisms. Neurosci Biobehav Rev 85:117–125. 28571877 10.1016/j.neubiorev.2017.05.019 PMC5708151

[B19] Ersche KD, Roiser JP, Abbott S, Craig KJ, Müller U, Suckling J, Ooi C, Shabbir SS, Clark L, Sahakian BJ, Fineberg NA, Merlo-Pich EV, Robbins TW, Bullmore ET (2011) Response perseveration in stimulant dependence is associated with striatal dysfunction and can be ameliorated by a D 2/3 receptor agonist. Biol Psychiatry 70:754–762. 10.1016/j.biopsych.2011.06.033 21967987

[B20] Faul F, Erdfelder E, Lang AG, Buchner A (2007) G*Power 3: a flexible statistical power analysis program for the social, behavioral, and biomedical sciences. Behav Res Methods 39:175–191. 17695343 10.3758/bf03193146

[B21] Fernando ABP, Economidou D, Theobald DE, Zou MF, Newman AH, Spoelder M, Caprioli D, Moreno M, Hipólito L, Aspinall AT, Robbins TW, Dalley JW (2012) Modulation of high impulsivity and attentional performance in rats by selective direct and indirect dopaminergic and noradrenergic receptor agonists. Psychopharmacology (Berl) 219:341–352. 10.1007/s00213-011-2408-z 21761147 PMC3249163

[B22] Ferster CB, Skinner BF (1957) Differential reinforcement of rate In: Schedules of reinforcement, pp 464–507. East Norwalk, CT: Appleton-Century-Crofts.

[B23] Groman SM, James AS, Jentsch JD (2009) Poor response inhibition: at the nexus between substance abuse and attention deficit/hyperactivity disorder. Neurosci Biobehav Rev 33:690–698. 18789354 10.1016/j.neubiorev.2008.08.008 PMC2728075

[B24] Groman SM, Lee B, London ED, Mandelkern MA, James AS, Feiler K, Rivera R, Dahlbom M, Sossi V, Vandervoort E, Jentsch JD (2011) Dorsal striatal D2-like receptor availability covaries with sensitivity to positive reinforcement during discrimination learning. J Neurosci 31:7291–7299. 10.1523/JNEUROSCI.0363-11.2011 21593313 PMC3114883

[B25] Groman SM, Lee B, Seu E, James AS, Feiler K, Mandelkern MA, London ED, Jentsch JD (2012) Dysregulation of D(2)-mediated dopamine transmission in monkeys after chronic escalating methamphetamine exposure. J Neurosci 32:5843–5852. 10.1523/JNEUROSCI.0029-12.2012 22539846 PMC3353813

[B26] Groman SM, James AS, Seu E, Tran S, Clark TA, Harpster SN, Crawford M, Burtner JL, Feiler K, Roth RH, Elsworth JD, London ED, Jentsch JD (2014) In the blink of an eye: relating positive-feedback sensitivity to striatal dopamine D2-like receptors through blink rate. J Neurosci 34:14443–14454. 10.1523/JNEUROSCI.3037-14.2014 25339755 PMC4205561

[B27] Groman SM, Smith NJ, Petrullli JR, Massi B, Chen L, Ropchan J, Huang Y, Lee D, Morris ED, Taylor JR (2016) Dopamine D3 receptor availability is associated with inflexible decision making. J Neurosci 36:6732–6741. 10.1523/JNEUROSCI.3253-15.2016 27335404 PMC4916249

[B28] Grottick AJ, Higgins GA (2002) Assessing a vigilance decrement in aged rats: effects of pre-feeding, task manipulation, and psychostimulants. Psychopharmacology (Berl) 164:33–41. 10.1007/s00213-002-1174-3 12373417

[B29] Haluk DM, Floresco SB (2009) Ventral striatal dopamine modulation of different forms of behavioral flexibility. Neuropsychopharmacology 34:2041–2052. 10.1038/npp.2009.21 19262467

[B30] Holroyd KB, Adrover MF, Fuino RL, Bock R, Kaplan AR, Gremel CM, Rubinstein M, Alvarez VA (2015) Loss of feedback inhibition via D2 autoreceptors enhances acquisition of cocaine taking and reactivity to drug-paired cues. Neuropsychopharmacology 40:1495–1509. 10.1038/npp.2014.336 25547712 PMC4397408

[B31] Idris NF, Repeto P, Neill JC, Large CH (2005) Investigation of the effects of lamotrigine and clozapine in improving reversal-learning impairments induced by acute phencyclidine and d-amphetamine in the rat. Psychopharmacology (Berl) 179:336–348. 10.1007/s00213-004-2058-5 15645224

[B33] Izquierdo A, Jentsch JD (2012) Reversal learning as a measure of impulsive and compulsive behavior in addictions. Psychopharmacology (Berl) 219:607–620. 22134477 10.1007/s00213-011-2579-7 PMC3249486

[B34] Janssen LK, Sescousse G, Hashemi MM, Timmer MHM, Ter Huurne NP, Geurts DEM, Cools R (2015) Abnormal modulation of reward versus punishment learning by a dopamine D2-receptor antagonist in pathological gamblers. Psychopharmacology (Berl) 232:3345–3353. 10.1007/s00213-015-3986-y 26092311 PMC4537492

[B35] Jentsch JD, Roth R (2000) Effects of antipsychotic drugs on dopamine release and metabolism in the central nervous system In: Neurotransmitter receptors in actions of antipsychotic medications (Lidow MS, ed). Boca Raton: CRC Press.

[B36] Jentsch JD, Pennington ZT (2014) Reward, interrupted: inhibitory control and its relevance to addictions. Neuropharmacology 76:479–486. 23748054 10.1016/j.neuropharm.2013.05.022 PMC4023480

[B37] Jentsch JD, Olausson P, De La Garza R, Taylor JR (2002) Impairments of reversal learning and response perseveration after repeated, intermittent cocaine administrations to monkeys. Neuropsychopharmacology 26:183–190. 10.1016/S0893-133X(01)00355-4 11790514

[B38] Jentsch JD, Ashenhurst JR, Cervantes MC, Groman SM, James AS, Pennington ZT (2014) Dissecting impulsivity and its relationships to drug addictions. Ann NY Acad Sci 1327:1–26. 10.1111/nyas.12388 24654857 PMC4360991

[B39] Keeler JF, Pretsell DO, Robbins TW (2014) Functional implications of dopamine D1 vs. D2 receptors: a “prepare and select” model of the striatal direct vs. indirect pathways. Neuroscience 282:156–175. 25062777 10.1016/j.neuroscience.2014.07.021

[B40] Koulchitsky S, Delairesse C, Beeken T, Monteforte A, Dethier J, Quertemont E, Findeisen R, Bullinger E, Seutin V (2016) Activation of D2 autoreceptors alters cocaine-induced locomotion and slows down local field oscillations in the rat ventral tegmental area. Neuropharmacology 108:120–127. 10.1016/j.neuropharm.2016.04.034 27130904

[B41] Kruzich PJ, Grandy DK (2004) Dopamine D2 receptors mediate two-odor discrimination and reversal learning in C57BL/6 mice. BMC Neurosci 5:12. 10.1186/1471-2202-5-12 15061865 PMC400732

[B42] Kruzich PJ, Mitchell SH, Younkin A, Grandy DK (2006) Dopamine D2 receptors mediate reversal learning in male C57BL/6J mice. Cogn Affect Behav Neurosci 6:86–90. 16869233 10.3758/cabn.6.1.86

[B43] Laughlin RE, Grant TL, Williams RW, Jentsch JD (2011) Genetic dissection of behavioral flexibility: reversal learning in mice. Biol Psychiatry 69:1109–1116. 10.1016/j.biopsych.2011.01.014 21392734 PMC3090526

[B44] Le Foll B, Gallo A, Le Strat Y, Lu L, Gorwood P (2009) Genetics of dopamine receptors and drug addiction: a comprehensive review. Behav Pharmacol 20:1–17. 10.1097/FBP.0b013e3283242f05 19179847

[B45] Lee B, Groman S, London ED, Jentsch JD (2007) Dopamine D2/D3 receptors play a specific role in the reversal of a learned visual discrimination in monkeys. Neuropsychopharmacology 32:2125–2134. 10.1038/sj.npp.1301337 17299511

[B46] Lee B, London ED, Poldrack RA, Farahi J, Nacca A, Monterosso JR, Mumford JA, Bokarius AV, Dahlbom M, Mukherjee J, Bilder RM, Brody AL, Mandelkern MA (2009) Striatal dopamine D2/D3 receptor availability is reduced in methamphetamine dependence and is linked to impulsivity. J Neurosci 29:14734–14740. 10.1523/JNEUROSCI.3765-09.2009 19940168 PMC2822639

[B47] Liao RM, Cheng RK (2005) Acute effects of d-amphetamine on the differential reinforcement of low-rate (DRL) schedule behavior in the rat: comparison with selective dopamine receptor antagonists. Chin J Physiol 48:41–50. 15973966

[B48] Lobarinas E, Falk JL (1999) Dose-dependent effects but not sensitization of DRL 45-s performance by oral d-amphetamine with cumulative- and repeated-dosing regimens. Behav Pharmacol 10:739–746. 10.1097/00008877-199912000-00005 10780289

[B49] Loos M, Staal J, Schoffelmeer ANM, Smit AB, Spijker S, Pattij T (2010) Inhibitory control and response latency differences between C57BL/6J and DBA/2J mice in a Go/No-Go and 5-choice serial reaction time task and strain-specific responsivity to amphetamine. Behav Brain Res 214:216–224. 10.1016/j.bbr.2010.05.027 20580749

[B50] Marinelli M, Cooper DC, Baker LK, White FJ (2003) Impulse activity of midbrain dopamine neurons modulates drug-seeking behavior. Psychopharmacology (Berl) 168:84–98. 10.1007/s00213-003-1491-1 12721782

[B51] Mehta MA, Swainson R, Ogilvie AD, Sahakian J, Robbins TW (2001) Improved short-term spatial memory but impaired reversal learning following the dopamine D(2) agonist bromocriptine in human volunteers. Psychopharmacology (Berl) 159:10–20. 10.1007/s002130100851 11797064

[B52] Milienne-Petiot M, Kesby JP, Graves M, van Enkhuizen J, Semenova S, Minassian A, Markou A, Geyer MA, Young JW (2017) The effects of reduced dopamine transporter function and chronic lithium on motivation, probabilistic learning, and neurochemistry in mice: modeling bipolar mania. Neuropharmacology 113:260–270. 10.1016/j.neuropharm.2016.07.030 27732870 PMC5148687

[B53] Morita M, Wang Y, Sasaoka T, Okada K, Niwa M, Sawa A, Hikida T (2016) Dopamine D2L receptor is required for visual discrimination and reversal learning. Mol Neuropsychiatry 2:124–132. 10.1159/000447970 27867937 PMC5109995

[B32] National Research Council (2011) Guide for the Care and Use of Laboratory Animals: Eighth Edition Washington, DC: The National Academies Press.

[B54] Paterson NE, Ricciardi J, Wetzler C, Hanania T (2011) Sub-optimal performance in the 5-choice serial reaction time task in rats was sensitive to methylphenidate, atomoxetine and d-amphetamine, but unaffected by the COMT inhibitor tolcapone. Neurosci Res 69:41–50. 10.1016/j.neures.2010.10.001 20934466

[B55] Pattij T, Janssen MCW, Vanderschuren LJMJ, Schoffelmeer ANM, van Gaalen MM (2007) Involvement of dopamine D1 and D2 receptors in the nucleus accumbens core and shell in inhibitory response control. Psychopharmacology (Berl) 191:587–598. 10.1007/s00213-006-0533-x 16972104

[B56] Quinn PD, Stappenbeck CA, Fromme K (2011) Collegiate heavy drinking prospectively predicts change in sensation seeking and impulsivity. J Abnorm Psychol 120:543–556. 10.1037/a0023159 21443288 PMC3128198

[B57] Robbins TW (2002) The 5-choice serial reaction time task: behavioural pharmacology and functional neurochemistry. Psychopharmacology (Berl) 163:362–380. 12373437 10.1007/s00213-002-1154-7

[B58] Robinson ESJ, Eagle DM, Economidou D, Theobald DEH, Mar AC, Murphy ER, Robbins TW, Dalley JW (2009) Behavioural characterisation of high impulsivity on the 5-choice serial reaction time task: specific deficits in “waiting” versus “stopping.” Behav Brain Res 196:310–316. 10.1016/j.bbr.2008.09.021 18940201

[B59] Sanchez-Roige S, Peña-Oliver Y, Stephens DN (2012) Measuring impulsivity in mice: the five-choice serial reaction time task. Psychopharmacology (Berl) 219:253–270. 10.1007/s00213-011-2560-5 22089700

[B60] Segal DS, Kuczenski R (1997) Effects of methylphenidate on extracellular dopamine, serotonin, and norepinephrine: comparison with amphetamine. J Neurochem 68:2032–2037. 9109529 10.1046/j.1471-4159.1997.68052032.x

[B61] Seu E, Jentsch JD (2009) Effect of acute and repeated treatment with desipramine or methylphenidate on serial reversal learning in rats. Neuropharmacology 57:665–672. 10.1016/j.neuropharm.2009.08.007 19703480 PMC2783924

[B62] Soares-Cunha C, Coimbra B, Sousa N, Rodrigues AJ (2016) Reappraising striatal D1- and D2-neurons in reward and aversion. Neurosci Biobehav Rev 68:370–386. 27235078 10.1016/j.neubiorev.2016.05.021

[B63] Spronk DB, Van Der Schaaf ME, Cools R, De Bruijn ERA, Franke B, Van Wel JHP, Ramaekers JG, Verkes RJ (2016) Acute effects of cocaine and cannabis on reversal learning as a function of COMT and DRD2 genotype. Psychopharmacology (Berl) 233:199–211. 10.1007/s00213-015-4141-5 26572896 PMC4700084

[B64] Stoffel EC, Cunningham KA (2008) The relationship between the locomotor response to a novel environment and behavioral disinhibition in rats. Drug Alcohol Depend 92:69–78. 10.1016/j.drugalcdep.2007.06.012 17997051

[B65] Uhl GR (2006) Molecular genetics of addiction vulnerability. NeuroRx 3:295–301. 10.1016/j.nurx.2006.05.006 16815213 PMC3593379

[B66] van der Schaaf ME, Fallon SJ, Ter Huurne N, Buitelaar J, Cools R (2013) Working memory capacity predicts effects of methylphenidate on reversal learning. Neuropsychopharmacology 38:2011–2018. 10.1038/npp.2013.100 23612436 PMC3746683

[B67] Van Gaalen MM, Brueggeman RJ, Bronius PFC, Schoffelmeer ANM, Vanderschuren LJMJ (2006) Behavioral disinhibition requires dopamine receptor activation. Psychopharmacology (Berl) 187:73–85. 10.1007/s00213-006-0396-1 16767417

[B68] Volkow ND, Morales M (2015) The brain on drugs: from reward to addiction. Cell 162:712–725. 10.1016/j.cell.2015.07.046 26276628

[B69] Voon V, Irvine MA, Derbyshire K, Worbe Y, Lange I, Abbott S, Morein-Zamir S, Dudley R, Caprioli D, Harrison NA, Wood J, Dalley JW, Bullmore ET, Grant JE, Robbins TW (2014) Measuring “waiting” impulsivity in substance addictions and binge eating disorder in a novel analogue of rodent serial reaction time task. Biol Psychiatry 75:148–155. 10.1016/j.biopsych.2013.05.013 23790224 PMC3988873

[B70] Wenger GR, Wright DW (1990) Behavioral effects of cocaine and its interaction with d-amphetamine and morphine in rats. Pharmacol Biochem Behav 35:595–600. 2339152 10.1016/0091-3057(90)90296-t

[B71] Yawata S, Yamaguchi T, Danjo T, Hikida T, Nakanishi S (2012) Pathway-specific control of reward learning and its flexibility via selective dopamine receptors in the nucleus accumbens. Proc Natl Acad Sci USA 109:12764–12769. 10.1073/pnas.1210797109 22802650 PMC3412032

